# Collapse of inferior vena cava during complex filter retrieval with consequent intra-procedural systemic hypotension and bradycardia: a case report

**DOI:** 10.1186/s42155-023-00361-2

**Published:** 2023-03-16

**Authors:** Tushar Garg, Izzet Altun, Bill S. Majdalany, Nariman Nezami

**Affiliations:** 1 Division of Vascular and Interventional Radiology, The Russell H. Morgan Department of Radiology and Radiological Science, Johns Hopkins University School of Medicine, Baltimore, MD USA; 2 Division of Vascular and Interventional Radiology, Department of Diagnostic Radiology and Nuclear Medicine, University of Maryland School of Medicine, Baltimore, MD USA; 3 Department of Radiology, University of Vermont Medical Center, Burlington, VT USA; 4 Experimental Therapeutics Program, University of Maryland Marlene and Stewart Greenebaum Comprehensive Cancer Center, Baltimore, MD USA

**Keywords:** Inferior vena cava, Filter, Retrieval, IVC collapse, Vasovagal reaction

## Abstract

**Background:**

Prolonged dwelling time of inferior vena cava (IVC) filters has been shown to increase the need for the use of complex IVC filter retrieval techniques. In this report, we describe a case of complex retrieval of an IVC filter with prolonged dwelling time, which was temporarily accompanied by severe bradycardia and hypotension.

**Case presentation:**

Fifty-nine**-**year-old male patient past medical history of morbid obesity, atrial fibrillation status post-ablation, obstructive sleep apnea, and end-stage renal disease presented for IVC filter retrieval 16 years after placement. When the IVC filter was covered by sheaths, and the IVC was temporarily collapsed and occluded, the patient developed severe bradycardia and hypotension without compensatory tachycardia. Contrast injection through the common femoral vein sheath showed complete occlusion of IVC while the IVC filter was covered by both sheaths, likely due to the embedment of the IVC filter in the wall by extensive fibrinous tissues. IVC filter was successfully retrieved, and the blood pressure and heart rate were improved immediately afterward. A large non-occlusive IVC thrombus was identified on the final venogram, which was aspirated using a mechanical thrombectomy device.

**Conclusion:**

Complex retrieval of IVC filters with prolonged dwelled time can result in acute severe bradycardia and hypotension due to vasovagal reaction, acute collapse, and occlusion of IVC in the setting of IVC filter embedment in the wall by extensive fibrinous tissues.

## Background

Percutaneous image-guided insertion of inferior vena cava (IVC) causes vena cava interruption and provides a therapeutic option for management of a sub-group of patients with high risk of venous thromboembolism. Although there remains a lack of clear consensus, IVC filter placement has been shown to reduce the risk of post-operative thromboembolic disease in high-risk patients undergoing bariatric surgery (Minocha et al. 2019). Considering the association between prolonged dwelling time and complications such as device fracture, migration, and deep venous thrombosis, currently, it is recommended to schedule a follow-up appointment for the filter retrieval once the indication for filter placement no longer exists. Prolonged dwelling time has been shown to increase the need for complex IVC filter retrieval techniques like forceps technique and laser-assisted removal (Desai et al. 2017). This case report describes intra-procedural bradycardia and hypotension that occurred during complex retrieval of an IVC filter with prolonged dwelling time.

## Case presentation

**Fig. 1:**
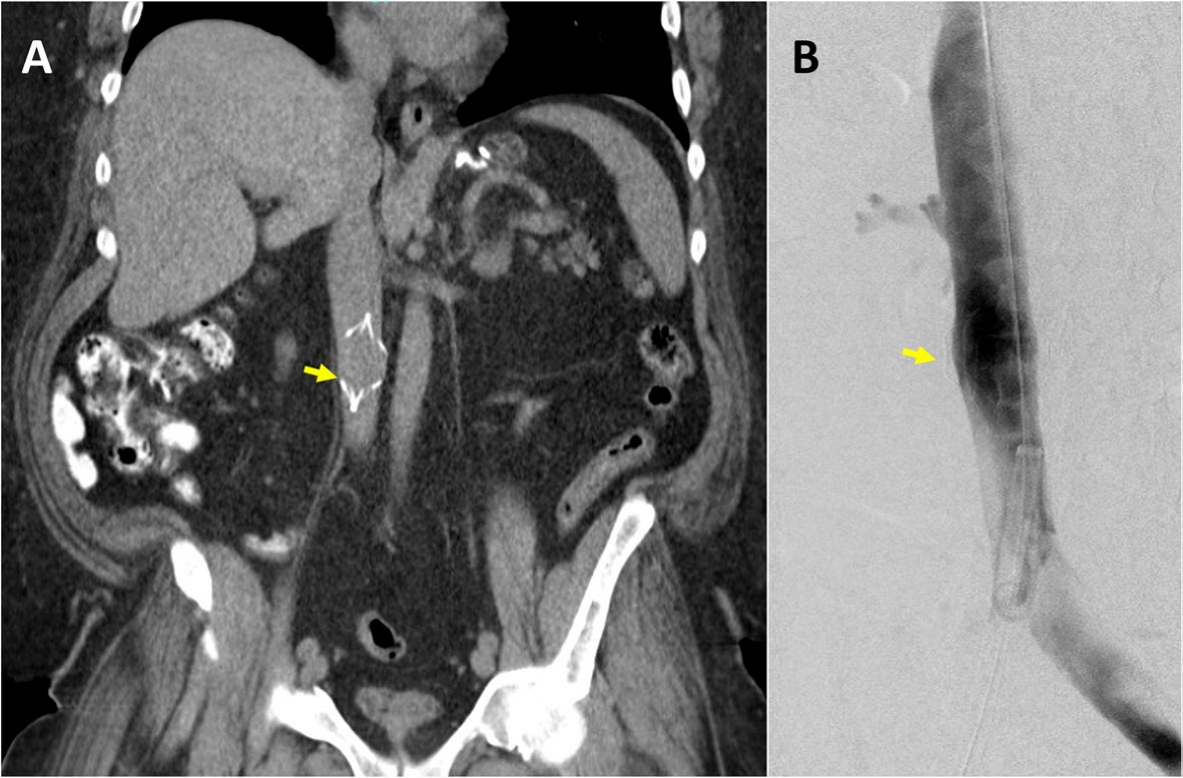
Pre-retrieval evaluation. **A** CT scan showed IVC filter (yellow arrow) embedded to the wall of IVC. **B** Initial injection through the pigtail catheter showed no thrombus within the IVC filter (yellow arrow)

**Fig. 2:**
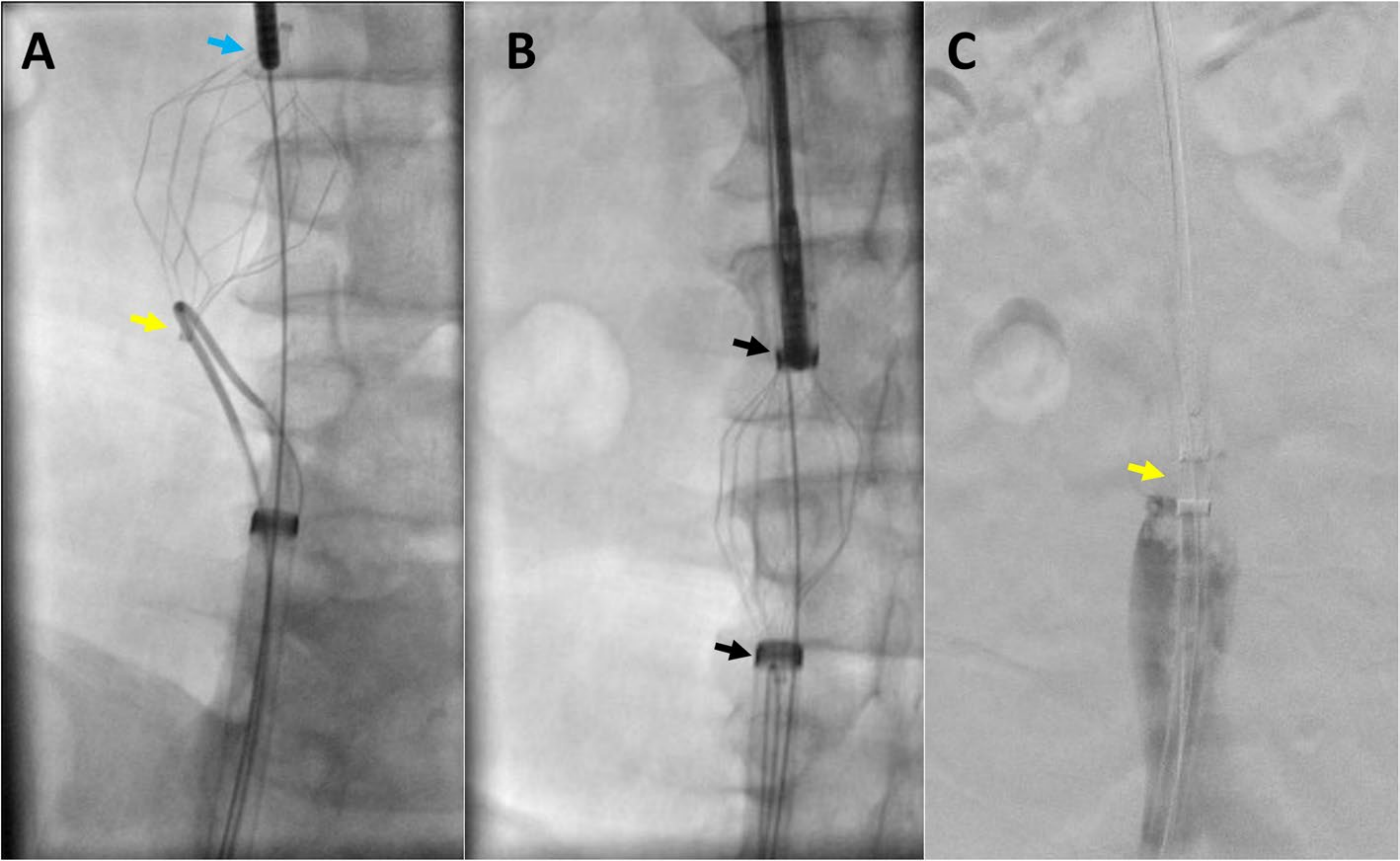
IVC filter retrieval. **A** While the upper end of the IVC filter is grabbed by endobronchial forceps (blue arrow), the Hangman technique was used to hook the lower end of the IVC filter (yellow arrow). **B** Both ends of the IVC filter are being covered by advancing sheath (black arrows) from opposite directions while holding tension on the IVC filter. **C** Once the IVC filter (yellow arrow) was completely collapsed and covered, sudden systemic hypotension is noted, and the contrast injection through the right CVF sheath shows IVC occlusion

**Fig. 3:**
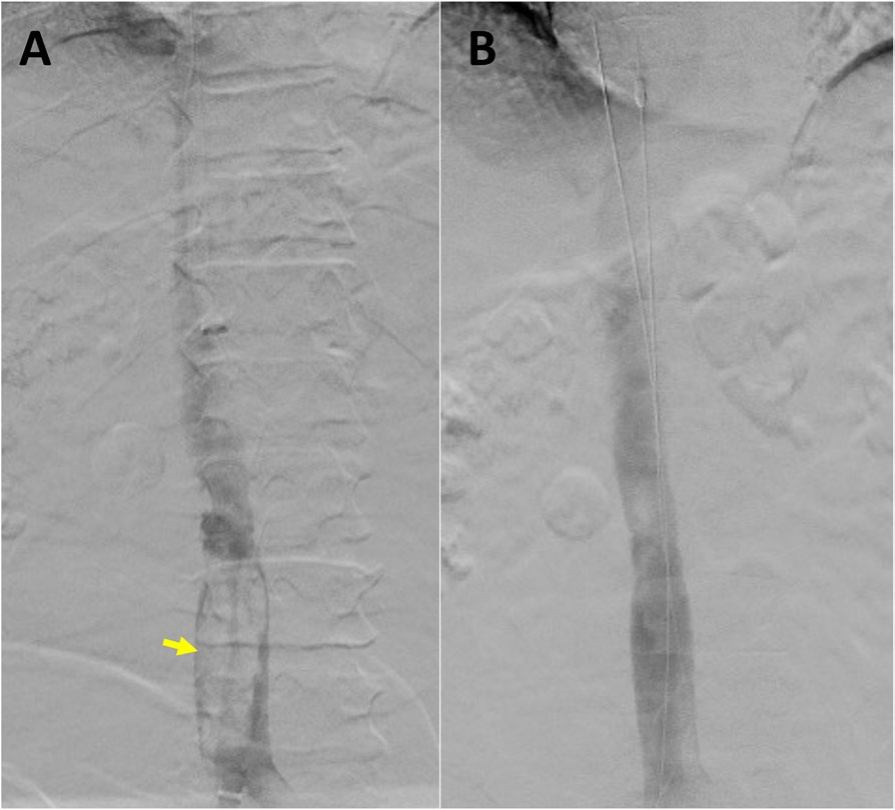
Post IVC filter retrieval. **A** IVC venogram after filter retrieval shows the presence of a large thrombus in the IVC (yellow arrow). **B** Complete resolution of the thrombus after starting IV heparin drip and preforming mechanical thrombectomy

This study was approved by the institutional review board, and consent was obtained from the patient. A 59-year-old male with a history of morbid obesity, atrial fibrillation status post ablation, obstructive sleep apnea (OSA) on continuous positive airway pressure (CPAP) and end-stage renal disease underwent placement of a permanent IVC filter (TRAPESE, Cordis Health, Miami Lakes, FL) 16 years ago before a bariatric surgery due to a high risk of thromboembolic disease at another health care center. However, the patient was lost to medical follow-up till recently, when the patient was undergoing a workup for a renal transplant. During the workup, an IVC filter with hooks embedded into the walls of the IVC was identified (Fig. 1A). The patient was referred to interventional radiology for IVC filter retrieval. The filter retrieval was performed under general anesthesia. Prior to procedure patient’s baseline blood pressure was around 120/60 mmHg. Access was gained to the right internal jugular vein (IJV) and right common femoral vein (CFV). A venogram of the IVC was then performed to assess IVC patency and anatomy as well as eventual presence of thrombus (Fig. 1B). A 18 Fr 55 cm sheath (Cook Medical, Bloomington, IN) was placed though the right IJV access and a 16 Fr 45 cm sheath (Cook Medical, Bloomington, IN) was advanced from the right CFV access (Cook Medical, Bloomington, IN) over an Amplatz wire (Boston Scientific, Marlborough, MA). The sheaths were positioned proximal and distal to the IVC filter hooks. A rigid endobronchial forceps (model 4162, Bryan, Woburn, MA) was then introduced through the right IJV access to remove the adhesions at the site of the upper end of the filter and grab the superior tip of the IVC filter. Next, an SOS Omni 1 catheter (Angiodynamics, Latham, NY) with the 0.035-inch stiff angled Glidewire (Terumo Interventional Systems, Somerset, NJ) was inserted through the right CFV access to utilize the Hangman technique, and the inferior part of the IVC filter was hooked (Fig. 2A). Once the sheaths were advanced over the IVC filter (Fig. 2B), and the filter was collapsed, the patient’s blood pressure suddenly dropped from 158/86 mm of Hg to 85/48 mm of Hg and the heart rate was dropped down to 48–52 beats/min range. The sudden drop in blood pressure and heart rate lasted for about 5 min, and contrast injection through the CFV sheath showed complete occlusion of the IVC when the filter was collapsed for retrieval (Fig. 2C). Soon after the IVC filter was uncovered by pulling back the sheaths, the patient’s heart rate and blood pressure were improved. The IVC filter was then completely retrieved. However, a large thrombus in the IVC was identified on the following IVC venogram (Fig. 3A). The patient was given a bolus dose of intravenous heparin (50 units per kg), and mechanical thrombectomy using FlowTriever System (Inari Medical, Irvine, California) was performed. The post-thrombectomy IVC venogram revealed complete aspiration of the thrombosis (Fig. 3B). After the procedure, the patient was transferred to the internal medicine floor for overnight observation and started on 1 mg/kg of subcutaneous enoxaparin sodium (Lovenox®) two times a day. During post-procedure monitoring, the patient’s blood pressure remained stable, and no symptoms of thromboembolic disease were seen.

## Discussion

In this case report, when the sheaths covered the IVC filter during the filter retrieval, there was a sudden drop in the systemic blood pressure without tachycardia. The patient was not on any beta blockers to explain the heart rate invariability. Two possible physiologic processes could explain this event. First, the sudden complete occlusion of the blood flow into the heart from the IVC collapse (Fig. 2C) could have decreased the cardiac preload before allowing the heart to compensate for hypotension by increasing the heart rate. However, the hypotension without tachycardia, in this case, continued for approximately 5 min. So, at least some increase in heart rate was expected, which did not happen. Second, there could have been sudden pooling of the blood into the abdominal (splanchnic) venous structures that triggered a vasovagal reaction leading to sudden systemic hypotension without a change in heart rate (Styczynski 2009). This reaction could be due to the pooling of the blood and the action of the splanchnic nerves supplying the IVC that act on the beta-2 receptors (Tucker 2022). The second mechanism seems the most likely etiology, given the lack of tachycardia in this scenario.

While the IVC filter was placed in this patient for prevention of thromboembolic even during bariatric surgery 16 years ago, IVC filter placement is no longer recommended for patients without known acute VTE who are undergoing major surgery, according to the current Society of Interventional Radiology Clinical Practice Guideline for Inferior Vena Cava Filters in the Treatment of Patients with Venous Thromboembolic Disease (Kaufman et al. 2020). Additionally, according to these guidelines, routine IVC filter removal can be performed in patients with indwelling IVC filters which are no longer at risk for thromboembolism (Kaufman et al. 2020). In a recent systemic review, including 37 studies and over 6800 patients, the mean retrieval rate was 34%, and the mean dwelling time was 72 days. The highest number of unanticipated complications from the use of filters were perforation, migration, and fracture, with the most common being filter migration and penetration into the wall of the vena cava, with 7% of these incidents occurring within the first 30 days after filter placement, and reported more frequently with prolonged use of the filter beyond 30 days (Angel et al. 2011). Although none of the above-reported complications occurred during this IVC filter retrieval, the procedure was complicated by the temporary development of unexpected hypotension and bradycardia during the last stage of IVC filter removal as well as thrombus formation immediately after IVC filter removal, most likely due to IVC endothelial injury and subendothelial exposure to blood flow.

## Conclusion

This case report highlights the acute severe bradycardia and hypotension as physiological changes which could occur due to vasovagal reaction, acute collapse, and occlusion of IVC in the setting of IVC filter embedment in the IVC wall by chronically developed extensive fibrinous tissues. The interventionalists performing IVC filter retrieval and anesthesiologists present during such procedures should know and possibly expect such a phenomenon during complex retrieval of IVC filters with prolonged dwell time.

## Data Availability

The datasets used and/or analyzed during the current study are available from the corresponding author on reasonable request.

## Abbreviations

IVC: Inferior vena cava
CFV: Common femoral vein
OSA: Obstructive sleep apnea
CPAP: Continuous positive airway pressure

## References

[CR1] Angel LF, Tapson V, Galgon RE, Restrepo MI, Kaufman J (2011). Systematic review of the Use of Retrievable Inferior Vena Cava Filters. J Vasc Interv Radiol.

[CR2] Desai KR, Laws JL, Salem R, Mouli SK, Errea MF, Karp JK (2017). Defining Prolonged Dwell Time: When Are Advanced Inferior Vena Cava Filter Retrieval Techniques Necessary?: An Analysis in 762 Procedures. Circ: Cardiovasc Interv..

[CR3] Kaufman JA, Barnes GD, Chaer RA, Cuschieri J, Eberhardt RT, Johnson MS (2020). Society of Interventional Radiology Clinical Practice Guideline for Inferior Vena Cava Filters in the treatment of patients with venous thromboembolic disease. J Vasc Interv Radiol.

[CR4] Minocha J, Smith AM, Kapoor BS, Fidelman N, Cain TR, Caplin DM (2019). ACR appropriateness Criteria® Radiologic management of venous thromboembolism-inferior Vena Cava Filters. J Am Coll Radiol.

[CR5] Styczynski G (2009). Dilated Inferior Vena Cava in Young adults with Vasovagal Syncope. Arch Intern Med.

[CR6] Tucker WD, Shrestha R, Burns B, Anatomy Abdomen and Pelvis, Inferior Vena Cava. In: StatPearls. Treasure Island (FL): StatPearls Publishing; 2022 [cited 27 June 2022]. Available from: http://www.ncbi.nlm.nih.gov/books/NBK482353/29493975

